# Editorial: Optimizing women’s health through exercise prescription and physiological assessments across life stages

**DOI:** 10.3389/fspor.2026.1893677

**Published:** 2026-07-21

**Authors:** Raphael Martins de Abreu, Étore De Favari Signini, Roberto Meroni, Oleksandr Romanchuk, Jordana Barbosa-Silva

**Affiliations:** 1 Department of Health, LUNEX University of Applied Sciences, Differdange, Luxembourg; 2 LUNEX ASBL Luxembourg Health & Sport Sciences Research Institute, Differdange, Luxembourg; 3 Department of Physiotherapy, Federal University of São Carlos, São Carlos, Brazil; 4 Department of Internal and Family Medicine, Lesya Ukrainka Volyn National University, Lutsk, Ukraine; 5 Department of Therapy and Rehabilitation, Ivan Boberskij Lviv State University of Physical Culture, Lviv, Ukraine; 6 Faculty of Business Management and Social Sciences, University of Applied Sciences, Hochschule Osnabrück, Osnabrück, Germany

**Keywords:** aging, exercise physiology, menopause, physical activity, sports medicine

This research topic aims to explore how exercise prescription and physiological assessment can be tailored to women's health across different life stages by bringing together research that addresses key factors that influence health, physical activity, and exercise responses, including menstrual health, reproductive factors, pregnancy, obesity, and pelvic floor function. This special issue includes eight articles examining elite and recreational athletes, women with overweight or obesity, and pregnant women. The contributions highlight the value of moving beyond generic recommendations toward more individualized models of assessment and intervention.

A few articles in this Research Topic focused on menstrual health in athletes, stressing that the menstrual cycle and hormonal contraceptive use are core dimensions of athlete monitoring. In a cross-sectional study by Crespo et al. of 228 elite Spanish basketball players, menstrual disturbances were commonly reported: 86% of respondents reported dysmenorrhea, 77% reported premenstrual syndrome symptoms, and 22% reported amenorrhea. These findings highlight the prevalence of menstrual health concerns among elite players and the need to incorporate menstrual history and screening into routine support systems for athletes. Additionally, in a study of 108 elite female athletes across seven sports, Badier et al. found that athletes often report more symptoms retrospectively than prospectively, suggesting that one-time or infrequent assessments may distort the symptom picture.

In a related contribution, Nolte et al. compared methods for assessing basal body temperature to detect ovulation in recreationally active women. The authors showed that intravaginal sensors for core body temperature can detect ovulation with acceptable accuracy compared to single-point sublingual, rectal, and external ear temperature measurements. Specifically, ovulation estimates derived from these thermometer-based methods, namely sublingual, rectal, and external ear thermometers, deviated by as much as 10 days from those obtained with the intravaginal sensor. These findings highlight that the value of physiological monitoring depends strongly on measurement validity, particularly when these data are used to support individualized exercise programming. For example, inaccurate ovulation detection could misclassify menstrual cycle phases, which could affect decisions about cycle-informed training load, recovery planning, and symptom monitoring.

Beyond menstrual monitoring, this research topic highlights how exercise interventions can be an effective strategy to improve health and function in women across different stages of life, as shown by Sašek et al. In untrained women aged 37–55, both standard resistance training and a velocity-based protocol using a 10% velocity-loss threshold improved lower-limb power, muscular endurance, and dynamic stability after 6 weeks of intervention. In this study, velocity-based training required participants to perform lower-body exercises with maximal lifting intent, while barbell velocity was monitored in real time; each set was terminated when movement velocity decreased by 10% from the target velocity. Notably, the velocity-based approach produced superior gains in muscular power with fewer repetitions, while the standard approach yielded somewhat greater improvements in muscular endurance. This study demonstrates that the structure of resistance training programs can be optimized according to the specific outcomes desired.

Another study by Zhao and Liu explored the impact of sprint interval training, with and without caffeine supplementation, in women with overweight or obesity. After a 12-week intervention, both exercise groups showed significant improvements compared to controls, while the caffeine group showed more favorable responses in terms of body composition, muscle strength, cardiorespiratory fitness, fasting glucose, and adipokine-related outcomes. Although the authors acknowledged the modest effect sizes and limited sample size, these results highlight the feasibility of time-efficient high-intensity training in this population.

The life-stage perspective of this research topic is evident in the study by Song et al., which explored the pregnancy-focused intervention based on the Fogg Behavior Model in women with obesity or overweight. The behaviorally informed intervention was associated with lower gestational weight gain, higher physical activity levels, greater physical activity self-efficacy in mid-pregnancy, and greater physical activity knowledge later in pregnancy. This study is a useful reminder that optimizing exercise prescription goes beyond physiology; it also involves behavior change, motivation, and opportunity, which are factors that make physical activity achievable during periods of major physical and psychological transition.

This collection also broadens the discussion from performance and exercise dosage to health-related outcomes. In one study, Duan et al. conducted a cross-sectional analysis using 2013–2018 NHANES data from 2,360 women aged 20–45 to examine whether composite cardiovascular health indices were associated with self-reported infertility. Specifically, the authors evaluated Life's Essential 8, which includes diet, physical activity, nicotine exposure, sleep health, body mass index, blood pressure, blood glucose, and blood lipids, along with Life's Crucial 9, which adds psychological well-being to these components. Women with higher scores on both indices had lower odds of infertility; in fully adjusted models, those in the highest quartile of Life's Essential 8 and Life's Crucial 9 had substantially lower odds of infertility than those in the lowest quartile. This study reinforces the idea that movement behaviors, cardiometabolic health, and reproductive health should not be studied separately. For sports researchers, this means considering exercise as not only a tool for physical performance and/or body composition but also as part of a broader reproductive and preventive health framework. However, because the study was cross-sectional and infertility was self-reported, causal conclusions cannot be drawn. Further prospective and interventional studies are needed to determine whether improving composite cardiovascular health contributes to better fertility outcomes.

Finally, an opinion article by Bortolami and Rossettini on pelvic floor health in sports and physical activity contexts added an important discussion to this Research topic. The authors stressed that pelvic floor health should be explicitly considered in sports settings, noting that while physical exercise is essential to women's health across the lifespan, it can also aggravate pelvic floor symptoms when dysfunction is already present. Importantly, underexplored conditions, such as increased pelvic floor muscle tone, pain, coordination disorders, and pudendal neuralgia, should also receive attention. This contribution reframes pelvic floor health as part of individualized exercise decision-making and emphasizes that sports professionals should balance the benefits of exercise with symptom presentation and clinical reasoning.

This research topic highlights key priorities and gaps in the field. Women's health research in sports and active living should continue to move toward valid and feasible approaches to monitoring, particularly with regard to menstrual symptoms, hormonal contraceptive use, and cycle-related physiology. From an exercise prescription perspective, approaches should be more clearly individualized according to health status, life stage, and desired outcomes. Future studies would also benefit from better integration of physiological, clinical, and behavioral perspectives, using models that incorporate symptom tracking, valid assessment methods, functional outcomes, and strategies that support adherence. Additionally, mechanistic studies exploring how hormonal status, training load, energy availability, pelvic floor function, and cardiometabolic health interact across the lifespan are needed. Importantly, intervention research should prioritize outcomes that matter to women, such as quality of life, symptom burden, confidence, and participation. Overall, these articles show that optimizing women's health through exercise is not simply a matter of prescribing more physical activity but also necessitates better measurement, more refined programming, and a deeper understanding of women's lived physiological experiences in sports and daily life.

